# Spatio-temporal epidemic forecasting with graph-based transformer

**DOI:** 10.1186/s12942-026-00476-4

**Published:** 2026-05-31

**Authors:** Mahmoud Ezzat, Youssef Mohamed Malek, Tamer AbdelKader, Nagwa Badr

**Affiliations:** 1 Department of Information Systems, Computer and Information Sciences, Ain Shams University, Abbasya, Cairo, 11566 Egypt; 2 Faculty of Computer Science and Engineering, Galala University, Ain Sukhna, 43511 Egypt; 3 Department of Media Engineering and Technology, Faculty of Engineering, German University in Cairo, New Cairo, 11835 Egypt

**Keywords:** COVID-19, Graph convolutional network, Transformer, Attention mechanism, Spatio-temporal

## Abstract

Epidemic forecasting plays a vital role in modern public health. The COVID-19 outbreak underscored the critical need for accurate and responsive models. Recent spatio-temporal Graph Neural Network (GNN) models that integrate human mobility networks face challenges in fully capturing complex, non-linear temporal dynamics and long-range spatial dependencies. To bridge this gap, we introduce two novel spatio-temporal architectures that combine GNNs with Transformer-based temporal modeling: a local-attention-model, which restricts self-attention to temporally adjacent windows of the same node, and a global-attention-model, which leverages full sequence-wide attention across all nodes to capture long-range dependencies. We benchmark our approaches against Persistence, Graph Convolutional Recurrent Network (GCRN) and Graph WaveNet baselines using two real-world datasets from Spain and Brazil. Our models show competitive and superior performance across most metrics compared to recurrent and temporal convolution baselines. The Linear Temporal Graph Convolutional Network (LinearTGCN) variant achieves the best Symmetric Mean Absolute Percentage Error (SMAPE) 24.74% and Mean Directional Accuracy (MDA) 72.52% on Spain dataset, outperforming the full attention-models. While, in Top-40 cities subset of Brazil dataset, local-attention-model slightly matches or outperforms the compared baselines with RMSE (3873.63) and SMAPE (83.47%). Our experiments demonstrate that simple linear models can match or exceed Transformers on structured time series, while Transformers show a great performance on noise or unstructured datasets like Brazil dataset. We found that SMAPE values varies across models by only a few percentage points, while the models are significantly better at directional prediction than the Persistence baseline.

## Introduction

In light of the growing frequency and severity of epidemic outbreaks such as COVID-19, the development of precise forecasting models has become a key for predicting future cases and providing neccessary public health responses. These models enable governments to inform public health policy and optimize the allocation of resources for disease control and prevention. Timely and reliable forecasts are truly essential, because early intervention can prevent a great number of deaths. When attempting to control an epidemic, one significant challenge involves predicting their future trajectory, highlighting the need for time series analysis within the field of epidemiology, where we utilize historical infection data to model and forecast the future outbreaks. Traditional statistical models, such as the Susceptible-Infected-Recovered (SIR) and Susceptible-Exposed-Infected-Recovered (SEIR) frameworks, have provided a basis for understanding disease transmission dynamics and highlighted the value of mathematical formulations for epidemic forecasting. However, these models often struggle to capture the complex, non-linear, and dynamic nature of epidemic spread, especially when handling with large volumes of temporally and spatially mobility and epidemiological data. To address these limitations and meet the evolving needs of epidemic prediction, researchers have increasingly turned to advanced machine learning and Deep Learning (DL) methods.

Human mobility data has an essential role in modeling epidemic spread as it captures key dimensions of human movement for both individuals and groups. Insights derived from the analysis of these patterns provide clear comprehension of transmission dynamics and improve the forecasting of future outbreaks. Modeling human mobility remains challenging due to its non-linear and irregular nature, the presence of long-range dependencies in its sequential movement, and the heterogeneity and sparsity of data collected from different sources. These challenges have motivated the development of multi-modal models that integrate multiple data types for better understanding of movement dynamics. In this context, combining graph-based models with Transformer architectures has emerged as a promising strategy to tackle these complexities.

The adoption of Transformer models marks as a turning point in spatio-temporal modeling, offering significant improvements over traditional methods, particularly in the context of epidemic forecasting. Unlike conventional approaches that rely on fixed assumptions, Transformers use attention mechanisms to dynamically identify and weigh the most relevant input features, enabling the model to focus on the factors that most strongly influence disease spread. This capacity allows for a more comprehensive representation of origin-destination (OD) flows and facilitates the integration of diverse data sources and complex dependencies. Additionally, advanced DL techniques have been increasingly applied to OD flow modeling in various domains, demonstrating the development and versatility of modern analytical frameworks [1]. In particular, Transformer-based architectures have achieved state-of-the-art performance in forecasting [2] and have also been successfully applied to anomaly detection tasks [3]. These strengths make Transformer-based models highly suitable for addressing the complexities of epidemic prediction.

GNNs further advance the modeling of epidemic dynamics by capturing structured relational information and complex spatial dependencies [4]. When integrated with sequential temporal models, such as Recurrent Neural Networks (RNNs) and Transformers, GNNs enable the joint learning of spatial and temporal dynamics by aggregating signals from neighboring nodes and evolving node and edge features over time [5]. Also, GNNs have shown strong performance in general time series tasks such as forecasting, anomaly detection, classification, and imputation, underscoring their growing significance in spatio-temporal machine learning [6].

Our study is intentionally positioned as a *controlled comparison* that builds directly on a GCN+RNN baseline (e.g., GCRN-style models): we keep the spatial encoder family fixed (GCN) and vary only the temporal mechanism (RNN vs. Transformer). This isolates the contribution of attention-based temporal encoding under identical preprocessing, splits, and metrics, helping to clarify when and why attention helps for epidemic forecasting with mobility-aware graphs. We additionally compare against two peer-reviewed external baselines: the persistence forecast and Graph WaveNet [7]. While complementary innovations such as fully learned or dual-scale graphs can be layered on top, our focus on the temporal swap provides a clear, reproducible test of a central hypothesis raised by recent literature.

The design of our two models; Local Transformer-GCN (LTGCN) and Global Transformer-GCN (GTGCN), reflects a deliberate tradeoff. LTGCN prioritizes scalability and interpretability: by applying self-attention independently per node, it avoids the quadratic cost in the number of nodes, making it applicable to large national-scale graphs. GTGCN prioritizes global expressiveness: by treating every node–time pair as a token, it enables full spatio-temporal cross attention at the cost of $\mathcal {O}((NT)^{2})$ memory, restricting it to compact graphs. The presentation of both designs allows us to empirically characterize this tradeoff and determine when global reasoning is worth the computational overhead.

The main contributions of this work are summarized as follows:

- We propose two novel Transformer-GCN architectures: the Local Transformer-GCN (LTGCN) and Global Transformer-GCN (GTGCN).
- We conduct extensive experiments comparing our models to a strong GCRN baseline, persistence baseline and Graph WaveNet model on two real-world datasets, demonstrating the competitive performance of Transformer-based models in trend detection Mean Directional Accuracy (MDA) and magnitude calibration Symmetric Mean Absolute Percentage Error (SMAPE).
- We provide a comprehensive preprocessing pipeline, including normalization, to ensure data quality and enhance the generalization capability of our models.

The remainder of this paper is organized as follows: Section "Related Work" reviews the relevant literature on epidemic prediction. Section "Methodology" details the proposed LTGCN and GTGCN models, including their architectures, problem formulation, preprocessing pipelines, and evaluation metrics. Section "Experiments" describes the experimental setup and presents our results. Section "Discussion" discusses key findings, highlights strengths and limitations, and outlines future research directions. Finally, Section "Conclusion" concludes the paper.

## Related work

The emergence of data analytics and computational techniques has significantly revealed our ability to predict epidemics, highlighting the need for new solutions to predict the evolution of outbreaks and the transmission of diseases. Traditionally, epidemic prediction models have relied on more basic statistical methods such as SIR and its extension SEIR. To implement better interventions and responses, modified SEIR models have been developed to model the COVID-19 epidemic, analyze its dynamics, and provide a framework for using mobility data [8–10]. These compartmental models have some limitations, such as estimating transmission parameters based on static assumptions and detailed statistics that are costly and resource intensive to collect. The Global Epidemic and Mobility Model (GLEaM) incorporates both compartmental dynamics and real-world human mobility networks to capture the spatial dimension of infectious disease spread [11]. However, it still depends on predefined parameters and static mobility assumptions, which may not reflect real-time behavioral or policy changes [12].

Deep learning techniques have emerged as powerful tools in epidemic forecasting, integrating both spatial and temporal dynamics to improve predictive accuracy. Many researches integrate DL techniques with compartmental models to reduce data dependency while estimating transmission parameters, thus improving the accuracy of epidemic forecasting [13, 14]. Another hybrid approach is the Epi-DNNs model [15], which uses a neural network to express transmission parameters that are considered the coefficients of the compartmental model. Nadler et al. [16] integrate the Susceptible-Infected-Recovered-Dead (SIRD) model with the Long Short-term Memory (LSTM) neural network to estimate time-varying parameters during outbreaks and leverage LSTM’s capacity for learning complex temporal patterns, thus improving the accuracy of epidemic prediction.

To overcome the limitations of hybrid and traditional compartmental models, researchers turned to use DL sequence models such as RNNs and their variants, LSTM and Gated Recurrent Units (GRUs). Due to their ability to avoid vanishing gradients in long sequences and learn long-term dependencies, LSTM-based models have become the most popular among DL sequence models in the domain of epidemic forecasting [17]. Shastri et al. [18] conduct a comparative case study of COVID-19 cases in India and USA based on LSTM variants; Stacked LSTM, Bi-directional LSTM and Convolutional LSTM. According to their findings, Convolutional LSTM outperformed the other variants in terms of prediction accuracy. In [19], the authors demonstrated that Convolutional Neural Networks (CNN) and Multivariate CNN outperformed LSTM and GRU models when forecasting with very few features and less amount of historical data. Otherwise, another study [20] showed that LSTM outperformed CNN and Multilayer Perceptron (MLP) while forecasting COVID-19 cases in Egypt for a week and a month ahead. MLP models have been effectively used to predict patient outcomes, especially in COVID-19 cases where clinicians need rapid responses. For example, Gao et al. [21] used MLPs to predict patient outcomes such as mortality risk and length-of-stay from Electronic Health Record data in Intensive Care Unit. Although MLPs lack the sophisticated attention mechanisms found in transformer structures, they present a practical alternative in real-time epidemic management where computational resources are limited and datasets are smaller. These models do not adequately incorporate spatial dependencies, which are essential components in the accurate modeling of infectious disease transmission. Furthermore, they may exhibit a deficiency in interpretability, which creates challenges in extracting valuable insights for policy-making or epidemiological research.

GNNs have gained significant traction in spatio-temporal modeling owing to their power in capturing complex and long-range spatial relationships and dependencies. The Graph Attention-based Spatial Temporal (GAST) model, for example, utilizes graph attention networks (GATs) to simulate epidemic dynamics, showcasing enhanced predictive performance for the spread of both influenza and COVID-19 [22]. Likewise, the Metapopulation-based Spatio-Temporal Attention Network (MPSTAN) integrates multi-patch epidemiological insights into a spatio-temporal framework, thereby increasing forecasting precision by dynamically characterizing inter-patch interactions [23]. The nodes of a graph are represented differently according to the modeling approach utilized in epidemic forecasting. In temporal modeling, each node represents a specific time step, capturing the temporal dynamics of the epidemic. Jin et al. [24] provide a comprehensive taxonomy of architectures and applications for recent progress on spatio-temporal graph neural networks (STGNNs) for predictive learning in urban computing. For instance, Li et al. [5] proposed an integrated model between GCN and Transformer, where a Transformer encodes temporal information, and GCN decodes it to capture spatial dependencies. While in spatial modeling, each node corresponds to a geographical region. Edges represent mobility connections between regions [25], and node features include time series data of infection rates [26]. To address the challenge of limited training data, they utilize a meta-learning approach to transfer knowledge between countries [25]. Duarte et al. [27] combined GCN-based encoders and recurrent temporal models to improve the accuracy of forecasting epidemic spread using a Brazilian intercity mobility network. Although these models have been applied successfully to epidemic time series forecasting, their reliance on recurrent architecture imposed limitations such as adversity in capturing long-range temporal dependencies, their limited flexibility in learning global attention across nodes and time steps, and their poor scalability due to the sequential computation.

Recent advances in spatio-temporal forecasting have produced sophisticated graph neural architectures that effectively capture complex dynamic dependencies. For adaptive graph learning, PSTCGCN [28] introduce parameterized graph structures that learn dynamic spatial correlations and use an effective temporal causal convolution component to capture local and global correlations sequentially. A model like Auto-DSTSG [29] refines the mechanism for extracting features across space and time simultaneously based on automated synchronous graph structure. Although developed for traffic forecasting, the core architectural innovations in these models align with three themes that our design incorporates as follows: (i) attention-driven temporal modeling to handle long-range, irregular dynamics; (ii) graph adaptivity (or compatibility with adaptive maps) to reflect evolving inter-regional connectivity; and (iii) multi-scale reasoning to reconcile local fluctuations with broader importation-driven trends.

Furthermore, Transformer architectures have demonstrated considerable efficiency in modeling long-term temporal dependencies via self-attention mechanisms, particularly when integrated with learnable positional encodings. Shao et al. [30] provide a large-scale benchmark for Multivariate Time Series Forecasting (MTSF) highlighting how temporal stability and spatial heterogeneity strongly influence model selection. They demonstrate that Transformers with their variants are recognized for their effectiveness in MTSF. Nevertheless, Transformers generally fail to incorporate spatial structures and inter-node interactions unless they are explicitly adapted for such purposes. Therefore, in this paper, we address this gap by proposing two novel variants of spatio-temporal Transformer architectures designed to capture epidemic dynamics over mobility graphs; LTGCN, where each city applies a Transformer encoder to its own historical case sequence independently, and GTGCN, where the Transformer is applied over a flattened sequence of all node-time tokens, enabling full global attention across both space and time. The local variant offers strong scalability and faster training, while the global variant excels in capturing long-range spatio-temporal interactions when computationally feasible. Our proposed Transformer–GCN variants inherit these strengths by preserving graph-based spatial aggregation while replacing the recurrent temporal stack with attention, thereby enabling selective, context-dependent temporal integration without discarding the inductive bias of spatial message passing.

## Methodology

To forecast COVID-19 cases across regions, we design a spatio-temporal DL pipeline grounded in graph-based representations of human mobility and standardized epidemic trajectories. This section details our formal problem definition, dataset construction, preprocessing pipeline, architectural components and Training & Evaluation steps.

### Problem definition

We address the task of short-term regional-level epidemic forecasting by modeling the spatio-temporal dynamics of COVID-19 using both historical case counts and human mobility data. Given a graph of mobility interactions and recent case trajectories, the objective is to predict the number of new cases in each region for the next day. We additionally compare against two peer-reviewed external baselines: the persistence forecast and Graph WaveNet.

Let $N$ be the number of nodes (regions), $T$ the historical input length, $F$ the number of case features per node ($F = 1$: daily new cases), and $H = 1$ the forecast horizon. The input tensor is $\textbf{X} \in \mathbb {R}^{B \times T \times N \times F}$, where $B$ is the batch size, and the target is $\hat{\textbf{Y}} \in \mathbb {R}^{B \times N \times H}$. The spatial structure is encoded as a directed weighted mobility graph $G = (V, E)$ with $|V| = N$ nodes and edge weights $w_{ij}$ representing average weekly mobility between regions $i$ and $j$. Static node attributes $\textbf{Z} \in \mathbb {R}^{N \times D}$ (e.g., normalized population, centrality scores) are available at graph construction time but do not vary across time steps. The learning task is to approximate a function $\mathcal {F}: (\textbf{X}, G, \textbf{Z}) \rightarrow \hat{\textbf{Y}},$, which outputs region-wise forecasts of new case counts across the forecast horizon.

### Datasets

We evaluated our models on COVID-19 cases and human mobility datasets from Brazil and Spain. The Brazilian graph includes 5, 385 cities and $\sim$21k edges (after backbone filtering), built from intercity vehicle flow data [31] and normalized using 2022 census population. The Spanish dataset includes province-level mobility from Ministry of Transport and Sustainable Mobility (MITMA) [32] and COVID-19 cases from Datadista [33], scaled by provincial population. In both cases, we construct directed and weighted graphs representing weekly flows.

### Data preprocessing

Accurate spatio-temporal forecasting depends on well-structured temporal signals and informative spatial graph representations. To ensure data quality and consistency, we apply a unified preprocessing pipeline to both the Brazil and Spain datasets, which include COVID-19 case counts and inter-regional mobility flows. This pipeline encompasses data cleaning, normalization, and backbone graph filtering, following the methodology established in our baseline study [27].

#### Brazil dataset

The Brazilian dataset comprises daily COVID-19 reports from over 5,000 municipalities (2020–2023). To ensure quality, we filtered cities with over 40 days of inactivity, extreme negative values (e.g., $<-200$), or statistical outliers exceeding $\mu + 10\sigma$ in 30-day windows. This yielded a cleaner subset of 1,305 cities for model training.

##### Normalization

We merged multi-year case files and clipped negatives to zero. To prevent nonstationarity from distorting z-score normalization, a log1p transform is applied to Brazilian case counts prior to z-scoring, accounting for the multi-order of magnitude variation across epidemic waves. Two normalization strategies were applied:

- **Per-capita scaling:** Cases per 100,000 people.
- **Z-score normalization:** Applied per city to standardize temporal dynamics.

##### Mobility graph

Using the intercity flow data [31], we constructed a weighted mobility graph and applied the disparity backbone algorithm [34] to retain statistically significant edges, following the approach adopted in the GCRN baseline study [27]. Edges with $p_{ij} < 0.01$ (from the disparity backbone [34]) or among the top-5 strongest connections per node were retained to maintain robust local connectivity. The graph was encoded using PyTorch Geometric [35] with node features including population and centrality.

#### Spain dataset

For Spain (52 provinces), we applied similar steps. Negative case values were clipped and standardized via Z-score normalization. The population was scaled with min-max normalization.

##### Mobility graph

The weekly travel matrices from MITMA [32] were cleaned, scaled, and filtered with the same $p_{ij}$-based backbone strategy. The final sparse graph encoded normalized travel volumes between provinces.

These pipelines ensured reliable, scale-invariant inputs and interpretable graph structure for downstream learning.

### Model architectures

We introduce three architectures of increasing expressiveness. GCRN "GCRN Baseline (Recurrent GCN).GCRN Baseline (Recurrent GCN)" is our recurrent baseline, encoding spatial-temporal dependencies through GCN-parameterized GRU gates applied step by step. LTGCN "LTGCN (Nodewise Temporal Attention).LTGCN (Nodewise Temporal Attention)" replaces the recurrent encoder with a per-node Transformer, independently encoding each node’s history while fusing spatial context from a GCN encoder via a cross-attention mechanism that scales linearly with the number of nodes. GTGCN "GTGCN (Flattened Global Attention).GTGCN (Flattened Global Attention)" extends this to full global attention by treating every node–time pair as a token in a single sequence, enabling direct spatio-temporal reasoning at the cost of quadratic complexity.

#### GCRN baseline (recurrent GCN)

GCRN serves as our baseline model and is adapted from the architecture proposed by Duarte et al. [27]. It captures both spatial and temporal dependencies by integrating graph convolutional layers within a GRU framework, as originally introduced by Seo et al. [36] and further developed for spatio-temporal forecasting in works like Diffusion Convolutional Recurrent Neural Network (DCRNN) [37].

At each time step $t$, the model updates node-level hidden states $\textbf{H}_t \in \mathbb {R}^{B \times N \times H}$ based on the current input $\textbf{X}_t \in \mathbb {R}^{B \times N \times F}$, the previous hidden state $\textbf{H}_{t-1}$, and the mobility graph $G$. This update is defined as:

$$\textbf{H}_t = \text {GRUGCN}(\textbf{X}_t, \textbf{H}_{t-1}, G)$$

The GRUGCN operator uses gated mechanisms–reset, update, and candidate gates–each parameterized via graph convolutions (GCNConv). This approach has been widely applied in spatio-temporal tasks such as traffic flow [7, 37] and infectious disease forecasting [27, 38].

After processing the full sequence of length $T$, the final hidden state $\textbf{H}_T$ is passed through a feedforward layer with ReLU activation to produce predictions $\hat{\textbf{Y}} \in \mathbb {R}^{B \times N \times H}$, where $H$ is the forecast horizon (typically 1 day).

**Fig. 1:**
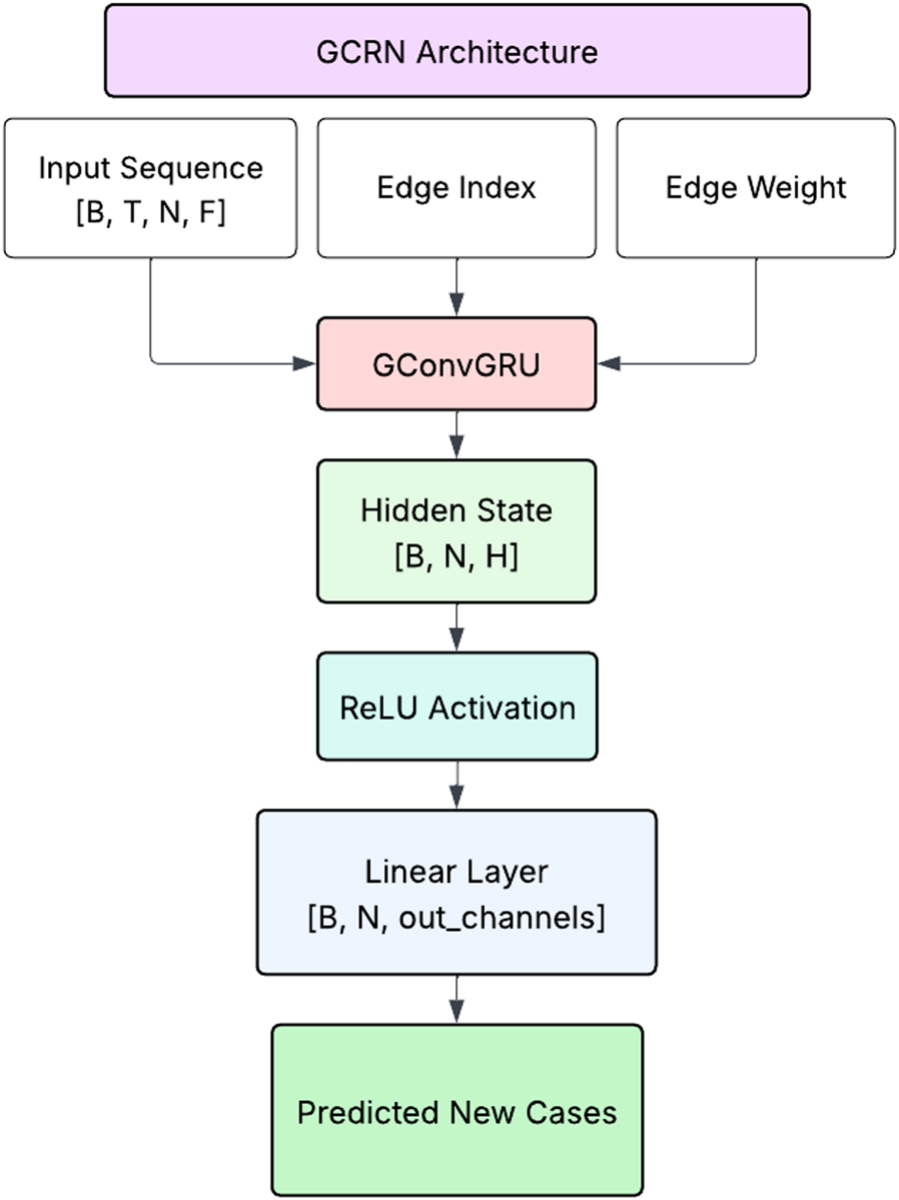
Architecture of the GCRN baseline. Each input frame passes through a GCN-parameterized GRU cell, with final hidden states used to generate per-node forecasts

We implement this architecture using a custom GConvGRUCell to support efficient batched training on sequences shaped $\textbf{X} \in \mathbb {R}^{B \times T \times N \times F}$. An overview of the architecture is shown in Figure 1.

Although effective, this model is inherently sequential and thus limited in modeling long-range temporal dependencies or capturing complex cross-regional interactions beyond localized graph propagation.

**Table 1:** Architecture and hyperparameters of the GCRN model used in our experiments

Component	Configuration
Input features	$in\_channels = 1$ (time-series feature: newCases)
Hidden state	$hidden\_channels = 288$ (Spain) - 144 Brazil
Output dimension	$out\_channels = 1$ (1-day ahead forecast per node)
Epochs	50
Optimizer	Adam ($lr=10^{-3}$)
Scheduler	ReduceLROnPlateau (patience=10, factor=0.7)

**Model layers**	
Gated graph cell	GConvGRUCell with three GCNConv gates per step: *z*, *r*, and $\tilde{h}$
Per-step graph ops	For each $t\in \{1,\dots ,T\}$: concat $[x_t, h_{t-1}]$; apply GCNConv-based gates; update $h_t$
Readout	$\text {Linear}(hidden\_channels \rightarrow out\_channels)$ after the final $h_T$
Nonlinearities	$\sigma$ for *z* and *r* gates; $\tanh$ for candidate; ReLU before final linear (optional)
Graph input	Shared $edge\_index$, optional $edge\_weight$ for all steps

As shown in Table 1, this configuration specifies the exact hyperparameters and architectural details that we adopt to train the GCRN baseline in our experiments. These settings ensure consistency across runs and align with prior work in spatio-temporal graph forecasting.

#### LTGCN (nodewise temporal attention)

**Fig. 2:**
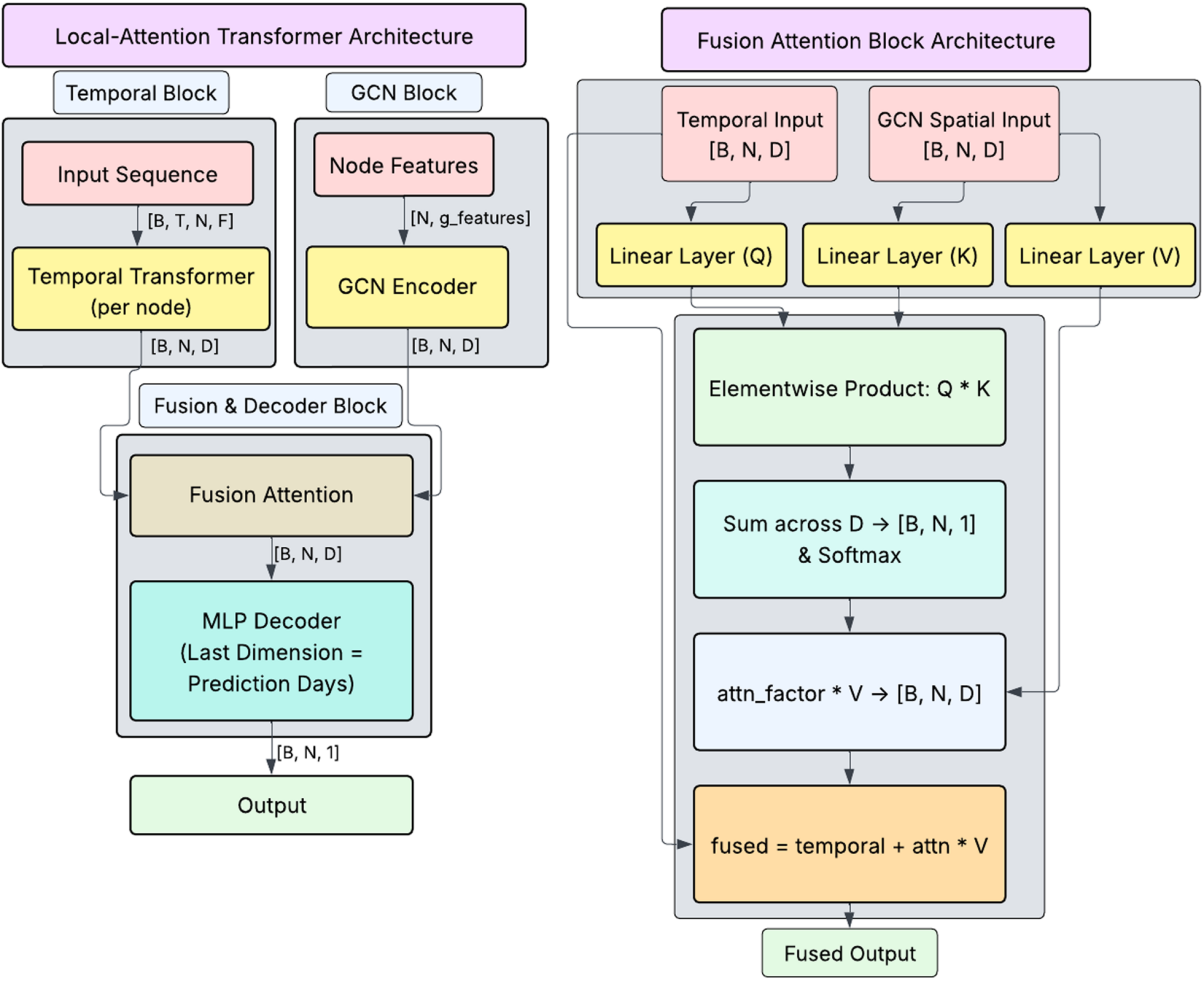
Architecture of the LTGCN model. Each node’s time series is encoded by a Transformer, while node embeddings are also computed globally via a GCN. A fusion attention mechanism integrates both temporal and spatial features per node

LTGCN applies a node-local Transformer; each node’s time series is encoded independently without cross-node attention during temporal processing, addressing the limitations of recurrent models in capturing long-range dependencies. This design leverages the Transformer’s self-attention mechanism [39] to enhance temporal expressiveness, building on recent advances in Transformer-based models for time series forecasting [2, 40–42]. An overview of the architecture and fusion mechanism is shown in Figure 2.

##### Temporal encoder

For each node $i$, we define the input sequence $X_i = [x_{i,1}, x_{i,2}, \dots , x_{i,T}] \in \mathbb {R}^{T \times F}$. This sequence is augmented with learnable positional encodings $P \in \mathbb {R}^{T \times d}$ and passed through a Transformer encoder:

1 $$\begin{aligned} \hat{X}_i = \text {TransformerEncoder}(X_i + P), \quad \hat{X}_i \in \mathbb {R}^{T \times d} \end{aligned}$$

This formulation captures intra-node temporal dependencies, inspired by nodewise Transformer applications in spatio-temporal forecasting such as Spatial-Temporal Transformer Network (STTNs) [43].

##### Spatial encoder

To capture global spatial context, a static GCN is applied to node-level features. The spatial representation for node $i$ is:

2 $$\begin{aligned} h_i^S = \text {GCN}(X_{\text {spatial}}, A)_i \end{aligned}$$

where $A$ denotes the adjacency matrix of the human mobility graph.

##### Fusion attention

To integrate temporal and spatial representations, we propose a local attention mechanism. First, a temporal summary $h_i^T \in \mathbb {R}^d$ is computed by extracting the last token of the Transformer output to preserve recency, as the final token has attended to all preceding positions as follows:

3 $$\begin{aligned} h_i^T = \hat{X}_{i,T} \end{aligned}$$

Having obtained the temporal summary $h_i^T$ from the sequential dynamics of node *i* and the spatial summary $h_i^S$ from its structural neighborhood via a per-node Transformer, we now fuse these two complementary representations to produce a unified node embedding. The softmax is applied over the *N* spatial representations, producing a scalar attention weight per node that gates its spatial contribution to the fused representation. The fused node representation $h_i^{\text {fused}}$ is then computed using attention:

4 $$\begin{aligned} q_i&= W_q h_i^T,\quad k_i = W_k h_i^S,\quad v_i = W_v h_i^S \end{aligned}$$

5 $$\begin{aligned} \alpha _i&= \text {softmax}_N(q \cdot k)_i \end{aligned}$$

6 $$\begin{aligned} h_i^{\text {fused}}&= h_i^T + \alpha _i v_i \end{aligned}$$

where $W_q, W_k, W_v \in \mathbb {R}^{d \times d}$ are learnable projection matrices. This attention mechanism selectively integrates spatial signals based on temporal dynamics, following joint encoder-fusion strategies commonly used in spatio-temporal forecasting models [44].

##### Model characteristics

By independently modeling each node’s time series, the model provides fine-grained, long-range temporal forecasts. However, this design does not explicitly model inter-node temporal interactions, which can be critical for capturing how regional influences propagate during epidemic outbreaks, as demonstrated in recent spatio-temporal attention models [45].

**Table 2:** Architecture and hyperparameters of the local spatio-temporal transformer

Component	Configuration
Input features	$in\_channels = 1$ (time-series feature: newCases)
Static node features	$graph\_feat\_dim = 1$ (e.g., population, centrality)
Hidden dimension	$trans\_hidden = 128$ (Spain),64 (Brazil), $nhead = 4$
Output dimension	$out\_channels = 1$ (1-day ahead prediction per node)
Epochs	50
Optimizer	Adam ($lr=3 \times 10^{-4}$ Spain, $lr=10^{-3}$ Brazil)
Scheduler	ReduceLROnPlateau (patience=10, factor=0.7)

**Model layers**	
Temporal encoder	Linear(1$\rightarrow$128) + Sinusoidal embedding + 1 Transformer layer ($nhead=4$)
Spatial encoder	2-layer GCN: GCNConv(1$\rightarrow$128)
Fusion	Attention-based fusion of temporal and spatial representations
Decoder	MLP: 128$\rightarrow$32$\rightarrow$1 (Spain), 64$\rightarrow$32$\rightarrow$1 (Brazil) with ReLU activations

As summarized in Table 2, this configuration defines the local spatio-temporal Transformer used in our experiments. The settings describe the precise hyperparameters and architectural choices employed for training, ensuring reproducibility and enabling a fair comparison against other baselines.

#### GTGCN (flattened global attention)

**Fig. 3:**
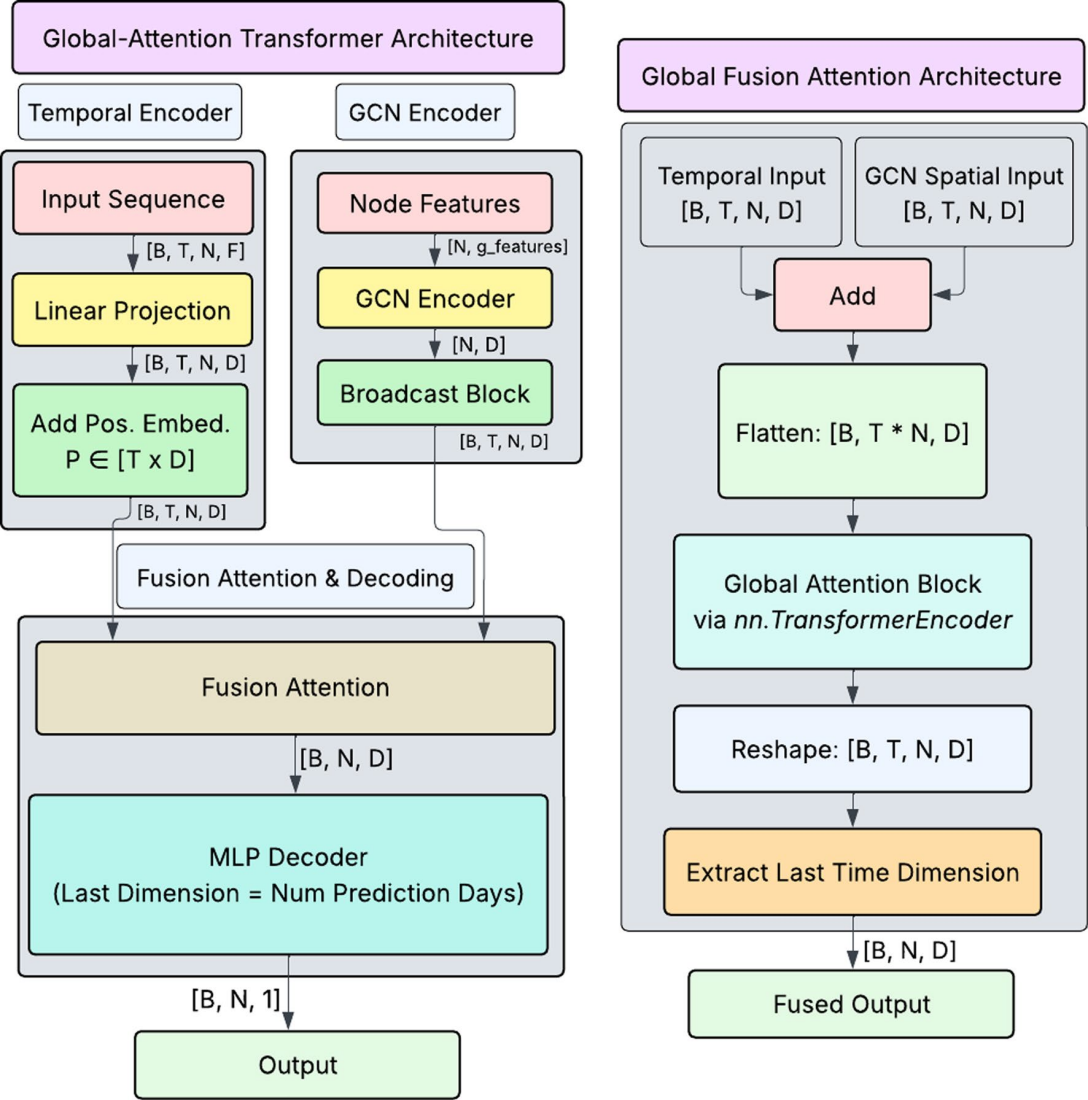
Architecture of the GTGCN model. Node features are enriched using a GCN encoder and fused with temporal embeddings before being flattened into a global spatio-temporal token sequence. A causal Transformer encoder enables attention across both space and time

Unlike LTGCN, GTGCN applies a global Transformer by flattening all *N* node × *T* timestep representations into a single token sequence, enabling any node at any timestep to attend to any other, and overcoming the locality constraints of recurrent and node-wise models. Unlike previous models that process each node independently or sequentially, this model treats each node-time pair as an individual token, allowing full spatio-temporal interaction across the entire input sequence. An overview is shown in Figure 3.

##### Temporal-spatial tokenization

The input $\textbf{X} \in \mathbb {R}^{B \times T \times N \times F}$ is first linearly projected to a hidden dimension $D$, producing $\textbf{X}_{\text {proj}} \in \mathbb {R}^{B \times T \times N \times D}$. Each node’s static features $\textbf{Z} \in \mathbb {R}^{N \times g}$ are processed via a GCN encoder:

7 $$\begin{aligned} &\textbf{S} = \textrm{GCN}(\textbf{Z}, A) \in \mathbb {R}^{N \times D}, \\&\quad \textbf{S}_{\text {seq}} = \text {Broadcast}(\textbf{S}) \in \mathbb {R}^{B \times T \times N \times D} \end{aligned}$$

This follows prior work that encodes node-level attributes with GCNs for spatial reasoning [37, 46].

##### Positional encoding and fusion

Sinusoidal positional encodings (deterministic, non-learnable) $\textbf{P} \in \mathbb {R}^{T \times N \times D}$ are added to encode time order. Similar temporal positional encoding strategies have been employed in time series Transformer models such as Informer [2]. The fused input becomes:

$$\textbf{X}_{\text {enc}} = \textbf{X}_{\text {proj}} + \textbf{S}_{\text {seq}} + \textbf{P}$$

##### Global transformer encoding and decoding

We flatten the 3D tensor to a sequence of shape $[B, T \cdot N, D]$, where each token corresponds to a specific node and time. A causal mask is applied to preserve auto-regressive structure. A multi-layer Transformer encoder captures interactions across all tokens:

8 $$\begin{aligned} \textbf{H} = \text {TransformerEncoder}(\text {Flatten}(\textbf{X}_{\text {enc}}), \text {mask}) \end{aligned}$$

Tokens are ordered row-major: all *N* nodes at timestep $t=0$, then $t=1$,$\ldots$,$t=T-1$. The causal mask enforces $M[i,j]=\text {True}$ (block) if and only if the timestep of token *j* is strictly greater than that of token $i: M[i,j] = [t_j> t_i]$. Tokens sharing the same timestep can attend to each other freely, enabling spatial reasoning within each step.

This design enables nonlocal information exchange across the entire spatio-temporal space, inspired by global self-attention strategies in graph representation learning [47] and spatio-temporal forecasting [43].

After reshaping back to $[B, T, N, D]$, we select the final timestep $\textbf{H}_T \in \mathbb {R}^{B \times N \times D}$ and pass it through MLP to predict case counts: $\hat{\textbf{Y}} = \textrm{MLP}(\textbf{H}_T)$

##### Complexity and scalability

The global attention mechanism operates over a flattened sequence of *TN* tokens per batch element, resulting in $\mathcal {O}((NT)^{2})$ memory for the attention matrix. With $T=14$ and $N=52$ (Spain), this produces $728^{2} \approx 530K$ attention entries per head — feasible. With $N=1,305$ (Brazil Cleaned), this grows to $(14\times 1305)^2 \approx 334M$ entries per head per batch element, requiring approximately 156 GiB at batch size 32 with 4 attention heads – infeasible on current hardware. GTGCN is therefore evaluated exclusively on the Top-40 Brazilian subset $(N=38)$. This is an architectural limitation that motivates the LTGCN design as the scalable alternative.

**Table 3:** Architecture and hyperparameters of the global spatio-temporal transformer

Component	Configuration
Input features	$input\_dim = 1$ (time-series feature: newCases)
Static node features	$gcn\_dim = 1$ (e.g., population, centrality)
Hidden dimension	$hidden\_dim = 128$ (Spain),64 (Brazil), $nhead = 4$
Attention heads	$nhead = 4$
Epochs	50
Transformer depth	$num\_layers = 1$ encoder layer
Optimizer	Adam ($lr=3 \times 10^{-4}$ Spain, $lr=10^{-3}$ Brazil)
Forecast dimension	$forecast\_dim = 1$ (per-node scalar)
Dropout	Transformer $=0.2$, Decoder MLP $=0.1$

**Model layers**	
Input projection	$\text {Linear}(1 \rightarrow 128)$
Spatial encoder	Two-layer GCN: $\text {GCNConv}(1\rightarrow 128)$
Temporal encoding	Sinusoidal (fixed), one vector per timestep.
Flattened tokens	Reshape to $[B, T\!\cdot \!N, 128]$ (Spain), $[B, T\!\cdot \!N, 64]$ (Brazil) with a causal attention mask
Transformer encoder	1 TransformerEncoderLayer ($d\_model=128$, $nhead=4$, dropout 0.2)
Decoder	MLP: 128$\rightarrow$64$\rightarrow$16$\rightarrow$1 (Spain), 64$\rightarrow$32$\rightarrow$16$\rightarrow$1 (Brazil) with ReLU, dropout 0.1
Output	[*B*, *N*, 1] per batch

As presented in Table 3, this configuration outlines the global spatio-temporal Transformer used in our study. The table specifies the key hyperparameters and architectural components adopted for training, providing clarity on the experimental setup and ensuring comparability with other models.

This globally attentive Transformer design offers full parallelism and holistic epidemic reasoning, enabling the model to learn complex spatio-temporal interactions such as regional contagion spread patterns, lagged dependencies, and nonlocal outbreaks.

#### Ablation variants

To assess the individual contribution of each key component, we conducted an ablation study by systematically removing or replacing one component at a time while keeping all other settings unchanged. Each variant was evaluated using the same 3-fold rolling cross-validation protocol on the Top 40 cities subset in Brazil dataset and the full dataset of Spain. Full systematic ablation variants are implemented as follows:

- **Temporal-only:** Self-attention on node time series, no GCN spatial branch.
- **Spatial-only:** GCN encoder + linear readout, no Transformer temporal branch.
- **No fusion attention:** Temporal + spatial concatenated, projected linearly (no cross-attention).
- **Linear temporal + GCN:** Linear lag encoder replaces self-attention (no attention at all).
- **Full LTGCN:** Our complete model.

### Training and evaluation

We adopt a consistent training pipeline and evaluation strategy across both datasets to ensure fair model comparison and statistical robustness. This includes a sliding window forecasting setup, standardized loss functions, and multiple performance metrics.

#### Sliding window forecasting strategy

To prevent data leakage, we apply a split-first strategy: the time series is partitioned at the date level into training (70%), validation (15%), and test (15%) segments before any windowing. Sliding windows of length T=14 are then extracted independently within each split, ensuring that no input window spans a split boundary and that no training sample includes test-period observations. While windows within a single split overlap by T-1 days (which is necessary to generate sufficient training samples), this overlap does not introduce leakage across splits. This procedure is strictly more conservative than post-hoc splitting of pre-windowed samples, which can cause target-day exposure through adjacent windows. Following prior work [27] that extends the work in [48], we use a fixed-length sliding window approach to generate training samples. For each region (node), we extract:

- **Input window:** 14 days of historical case data.
- **Output window:** 1-day-ahead prediction.
- **Feature:** Z-score normalized daily new cases.

#### Optimization setup

All models are trained using the Mean Squared Error (MSE) loss:

$$\mathcal {L} = \frac{1}{B \cdot N} \sum _{b=1}^{B} \sum _{n=1}^{N} \left( y_{bn} - \hat{y}_{bn} \right) ^2$$

This loss function is widely used in COVID-19 forecasting and spatio-temporal graph modeling due to its stability and interpretability. All models are matched to approximately equal parameter budgets within each dataset scale, and the parameter counts can be found in the repository to ensure fair comparison.

Weight decay (L2 regularization) is included for models requiring additional control over overfitting, as it is a standard strategy to improve generalization in Transformer-based and graph-based temporal models.

#### Evaluation metrics

To assess both prediction accuracy and trend correctness in COVID-19 forecasts, we adopt three complementary metrics: Root Mean Square Error RMSE, SMAPE, and MDA. These metrics have been widely adopted in epidemic modeling and spatio-temporal forecasting [27, 38, 41].

##### Root mean squared error (RMSE)

$$\text {RMSE} = \sqrt{\frac{1}{NT} \sum _{n=1}^{N} \sum _{t=1}^{T} \left( \hat{y}_{nt} - y_{nt} \right) ^2}$$

RMSE quantifies the absolute magnitude error and is commonly adopted as a primary metric in COVID-19 forecasting benchmarks [27, 38]. It penalizes larger deviations more heavily, making it useful for assessing peak prediction reliability and extreme case dynamics.

##### Symmetric mean absolute percentage error (SMAPE)

$$\text {SMAPE} = \frac{100}{T} \sum _{t=1}^{T} \frac{|\hat{y}_t - y_t|}{(|\hat{y}_t| + |y_t| + \epsilon )}$$

SMAPE measures relative error while addressing the limitations of MAPE when actual values are close to zero. This is particularly relevant in epidemic forecasting, where case counts often drop to low levels in tail phases. Prior work has used MAPE to assess COVID-19 forecasting accuracy [5, 49–51], but we opt for SMAPE to ensure robustness in low-value regimes and avoid instability from small denominators.

##### Mean directional accuracy (MDA)

$$\text {MDA} = \frac{1}{T - 1} \sum _{t=2}^{T} \mathbb {1} \left[ (\hat{y}_t - \hat{y}_{t-1})(y_t - y_{t-1})> 0 \right]$$

MDA evaluates whether the model correctly predicts the direction (increase or decrease) of future cases, regardless of magnitude. This is crucial in epidemiological models to anticipate trend shifts, surges, or declines.

##### Training and validation loss

In addition to the evaluation metrics above, we report *training and validation losses per epoch* by providing their loss curves for all models. Since validation loss often fluctuates due to stochastic optimization and subgraph batching, we report the Exponential Moving Average (EMA) of the validation loss to highlight the underlying trend. The EMA reduces noise by weighting recent epochs more heavily:

$$\text {EMA}_t = \alpha \, L_t + (1 - \alpha ) \, \text {EMA}_{t-1},$$

where $L_t$ is the raw validation loss at epoch *t*, and $\alpha \in (0,1]$ is the smoothing factor (we use $\alpha =0.3$ in our experiments). This smoothing highlights convergence behavior and mitigates spurious spikes, while still reflecting meaningful trend changes.

#### Reconstruction and visualization

Though models are trained on Z-score normalized inputs, we reverse the normalization for evaluation. Predictions $\hat{z}$ are reconstructed as:

$$\hat{Y}_{\text {original}} = {\left\{ \begin{array}{ll} \exp (\hat{z} \cdot \sigma + \mu ) - 1 & \text {when log1p is active} \\ \hat{z} \cdot \sigma + \mu & \text {otherwise} \end{array}\right. }$$

where $\mu$ and $\sigma$ are region-specific means and standard deviations. $\mu$ and $\sigma$ are estimated from the training partition only and applied without re-fitting to the validation and test sets, preventing data leakage into preprocessing. In the same line of [52], this approach facilitates the use of early stopping as a regularization technique to avoid overfitting, while also allowing assessment of model generalizability across a representative set of epidemic conditions.

#### Evaluation protocol

Each model is evaluated on a held-out test set comprising the final 15% of the time series, preceded by a 15% validation set used for hyperparameter selection and early stopping. The remaining 70% constitutes training data. Models are evaluated using 3-fold rolling-origin cross-validation. For fold 1; each subsequent fold shifts the test origin forward by an equal fraction of the remaining data while expanding the training set. This design ensures that at least one fold captures a peak epidemic wave in the test period, providing a conservative and realistic estimate of generalization. All reported metrics are computed in means ± standard deviation across the three folds for core models only (Persistence, GCRN, LTGCN and GTGCN), while ablations and additional baselines are reported at full data. All reported metrics (RMSE, SMAPE, MDA) evaluate point forecasts.

## Experiments

### Experimental setup

We compare recurrent and attention-based models for spatio-temporal forecasting across two settings. Our goal is to evaluate the impact of temporal encoders (GRU vs. Transformer) and spatial modeling (GCN), and to assess global vs. localized attention mechanisms under varying data quality. We compare our models against the following baselines: (i) Persistence—a naïve model that carries forward the last observed value, serving as a lower-bound skill reference standard in operational epidemic forecasting; (ii) GCRN—a recurrent GCN that integrates GCN-Conv gates inside a GRU cell; and (iii) Graph WaveNet—a model that combines adaptive graph convolution with dilated temporal convolutions.

We use 3-fold rolling-origin cross-validation to account for the non-stationarity of epidemic time series. Each fold uses an expanding training window and a fixed-length test window. In Section "Evaluation Metrics.Evaluation Metrics", we report RMSE, SMAPE, and MDA to measure absolute, relative, and directional performance, respectively.

***Model input/output overview***

In Table 4, we demonstrate the various input and output dimension shapes per component in the various models and explanatory notes per each component.

**Table 4:** Input and output dimensions of model components

Component	Input Shape	Output Shape	Notes
Raw Input	$X \in \mathbb {R}^{B\times T\times N\times F}$	—	Daily case features per node
Target	—	$\hat{Y} \in \mathbb {R}^{B\times N\times 1}$	Forecast horizon (1 day)
GCRN (baseline)	$X_t \in \mathbb {R}^{B\times N\times F}$	$H_t \in \mathbb {R}^{B\times N\times H}$	GRU cell with GCN parameterization
LTGCN – Temporal Encoder	$X_i \in \mathbb {R}^{T\times F}$	$\hat{X}_i \in \mathbb {R}^{T\times D}$	Nodewise Transformer per region
LTGCN – Spatial Encoder (GCN)	$Z \in \mathbb {R}^{N\times D}$	$h_i^S \in \mathbb {R}^{D}$	Static node features (e.g., population)
LTGCN – Fusion Attention	$h_i^T \in \mathbb {R}^{D}, h_i^S \in \mathbb {R}^{D}$	$h_i^{fused} \in \mathbb {R}^{D}$	Combines temporal + spatial features
GTGCN – Projection	$X \in \mathbb {R}^{B\times T\times N\times F}$	$X_{proj} \in \mathbb {R}^{B\times T\times N\times D}$	Linear map to hidden dim
GTGCN – Spatial Encoder (GCN)	$Z \in \mathbb {R}^{N\times g}$	$S \in \mathbb {R}^{N\times D}$	Node embeddings broadcast across *T*
GTGCN – Flattened Tokens	$X_{enc} \in \mathbb {R}^{B\times T\times N\times D}$	$Seq \in \mathbb {R}^{B\times (T\cdot N)\times D}$	Each node-time pair as a token
GTGCN – Transformer Encoder	$Seq \in \mathbb {R}^{B\times (T\cdot N)\times D}$	$H \in \mathbb {R}^{B\times (T\cdot N)\times D}$	Global spatio-temporal self-attention
GTGCN – Decoder (MLP)	$H_T \in \mathbb {R}^{B\times N\times D}$	$\hat{Y} \in \mathbb {R}^{B\times N\times 1}$	Final forecast per node

*B* = batch size, *T* = history length, *N* = number of nodes, *F* = input features, *H* = forecast horizon, *D* = hidden dimension

### Brazil experiments

Table 5 shows that Persistence, GCRN, GraphWaveNet, LinearTGCN and LTGCN models are trained on every Brazilian subset, while the GTGCN is restricted to the Top-40 cities because its $\mathcal {O}((NT)^{2})$ attention cost becomes prohibitive on larger graphs. This layout lets us quantify the effect of data cleaning and graph size, yet still compare all models on a tractable, high-priority subset.

**Table 5:** Coverage of models across Brazilian data subsets (✓ = model trained; — = not trained)

Subset	Persistence	GCRN	GraphWaveNet	LinearTGCN	LTGCN	GTGCN
Unfiltered (5,300+ cities)	✓	✓	✓	✓	✓	—
Cleaned (1,305 cities)	✓	✓	✓	✓	✓	—
Top 40 cities	✓	✓	✓	✓	✓	✓

#### Quantitative results on Brazilian dataset

Table 6 compares the forecasting performance of the competitive models on three subsets of the Brazilian dataset: the complete set of cities (5,300+), a cleaned high-quality subset (1,305 cities) and the top 40 most populous cities. In Table 7, we shows fold-by-fold (3-fold rolling cross-validation) comparison between Persistence, GCRN, LTGCN and GTGCN models using the evaluation metrics RMSE, SMAPE and MDA indicating avg ± std values on two subsets.

**Table 6:** Comprehensive evaluation of the competitive models across three data subsets: Top 40 cities, cleaned cities, and non-cleaned (all cities)

Subset	Metric	Persistence	GCRN	GraphWaveNet	LinearTGCN	LTGCN	GTGCN
**Top 40 cities**	RMSE	5482.07	3872.72	3878.59	3933.80	3873.63	3882.10
	SMAPE	99.50%	88.08%	100%	84.32%	83.47%	99.74%
	MDA	31.58%	47.39%	57.71%	49.68%	52.02%	43.30%
**Cleaned cities**	RMSE	1035.15	733.23	733.29	733.04	733.29	Not run
	SMAPE	108.68%	95.22%	95.46%	93.54%	95.43%	Not run
	MDA	55.78%	37.85%	48.51%	37.50%	37.35%	Not run
**Full (all cities)**	RMSE	705.57	499.42	499.45	499.37	499.40	Not run
	SMAPE	101.72%	89.58%	89.63%	88.27%	89.11%	Not run
	MDA	74.60%	22.71%	22.68%	22.47%	23.09%	Not run

The full global attention over N=1,305 nodes is computationally infeasible (the attention matrix grows as $\mathcal {O}((NT)^{2})$)

**Table 7:** Fold-by-fold (3-fold rolling cross-validation) RMSE, SMAPE and MDA comparison indicating avg ± std on core models on two subsets of Brazil dataset

Subset	Model	RMSE(F1/F2/F3; avg ± std)	SMAPE(F1/F2/F3; avg ± std)	MDA(F1/F2/F3; avg ± std)
**Top 40 Cities**	Persistence	1302.92/519.95/5482.07; 2434.98 ± 2178.20	85.17%/86.00%/99.50%; 90.22% ± 6.57	13.55%/12.94%/31.58%; 19.36% ± 8.65%
	GCRN	1153.52/349.51/3872.72; 1791.92 ± 1507.52	79.84%/71.00%/88.08%; 79.64% ± 6.97	57.19%/58.01%/47.39%; 54.20% ± 4.83%
	LTGCN	1218.46/384.76/3873.63; 1825.62 ± 1487.62	79.79%/70.97%/83.47%; 78.08% ± 5.24	57.14%/58.07%/52.02%; 55.74% ± 2.66%
	GTGCN	1259.58/455.80/3882.10; 1865.82 ± 1462.99	91.51%/90.04%/99.74%; 93.77% ± 4.27	54.44%/53.42%/43.30%; 50.39% ± 5.03%
**Cleaned Cities**	Persistence	290.16/324.40/1035.15; 549.90 ± 343.41	99.66%/99.06%/108.68%; 102.47% ± 4.40	38.37%/41.65%/55.78%; 45.26% ± 7.55%
	GCRN	240.03/234.20/733.23; 402.48 ± 233.88	90.81%/81.52%/95.22%; 89.18% ± 5.71	47.63%/47.11%/37.85%; 44.20% ± 4.50%
	LTGCN	251.92/235.47/733.29; 406.90 ± 230.89	97.26%/83.26%/95.43%; 91.98% ± 6.22	47.47%/46.52%/37.35%; 43.78% ± 4.56%
	GTGCN	Not run	Not run	Not run

**Fig. 4:**
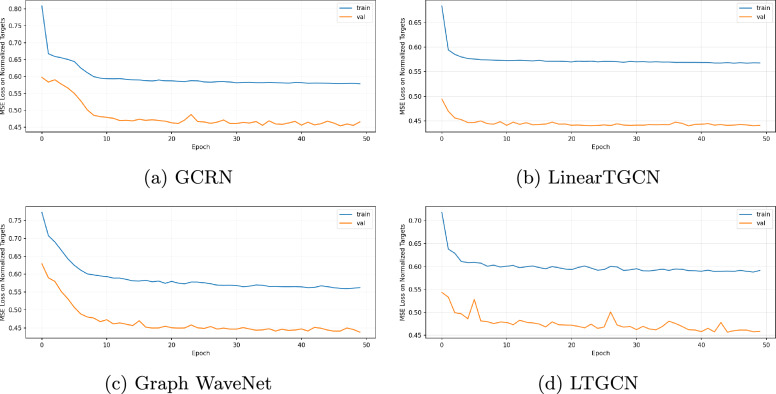
Training & validation loss graphs on the cleaned Brazilian subset for the comparable models using 50 epochs

**Fig. 5:**
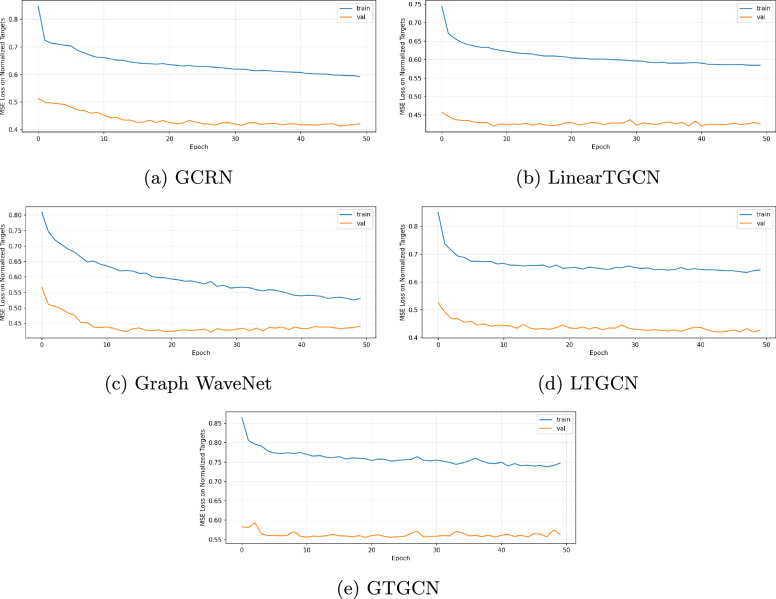
Training & validation loss graphs on the top 40 cities Brazilian subset for the comparable models using 50 epochs

Figure 4 shows the training and validation dynamics of the comparable models over 50 epochs on the cleaned Brazilian subset, while Figure 5 demonstrates the training and validation curves on the Top40 cities subset of Brazil dataset.

### Spain experiments

With only 52 provinces and consistent data quality, we train all model types (Persistence, GCRN, Graph WaveNet, LinearTGCN, LTGCN, and GTGCN) on the full Spanish dataset. This serves as a robustness evaluation under cleaner epidemiological conditions and provides insight into attention scope vs. performance trade-offs using a structured dataset.

#### Quantitative results on Spanish dataset

**Fig. 6:**
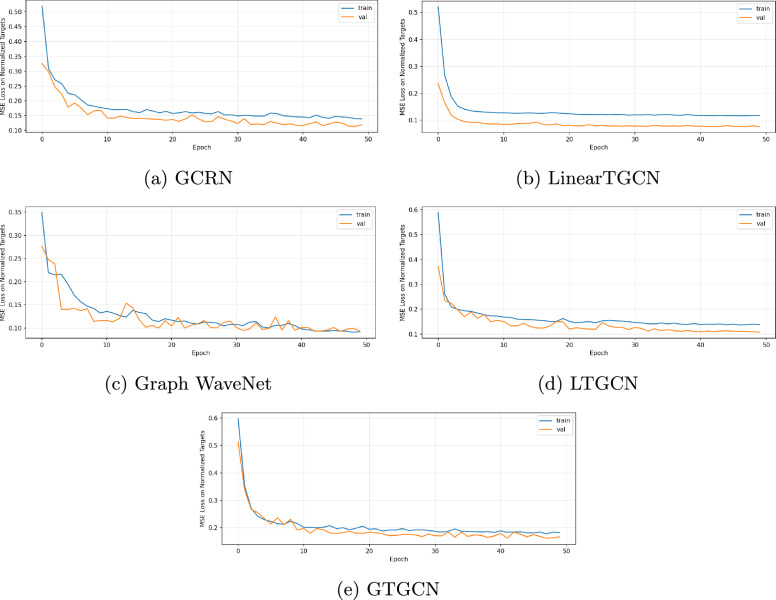
Training & validation loss graphs on the Spain dataset the comparable models using 50 epochs

Table 8 reports forecasting performance on the Spanish dataset for all competitive models which are evaluated using RMSE, SMAPE, and MDA on the full Spanish dataset. In Table 9, we shows fold-by-fold (3-fold rolling cross-validation) comparison between Persistence, GCRN, LTGCN and GTGCN models using the evaluation metrics RMSE, SMAPE and MDA indicating avg ± std values. Figure 6 demonstrates the training and validation dynamics of the comparable models over 50 epochs for all provinces in Spain dataset. Table 12 reports the attention complexity, batch size and average epoch time of each model on Spain dataset.

**Table 8:** Comparison of competitive models on the full Spanish dataset in terms of RMSE, SMAPE and MDA

Models	RMSE	SMAPE	MDA
Persistence	1035.96	34.00%	0.98%
GCRN	1422.58	38.76%	65.27%
Graph WaveNet	1670.83	40.28%	67.97%
LinearTGCN	682.92	24.74%	72.52%
LTGCN	1763.79	46.52%	64.18%
GTGCN	1838.79	51.72%	63.55%

**Table 9:** Fold-by-fold (3-fold rolling cross-validation) RMSE, SMAPE and MDA comparison indicating avg ± std on core models on Spain dataset

Model	RMSE(F1/F2/F3; avg ± std)	SMAPE(F1/F2/F3; avg ± std)	MDA(F1/F2/F3; avg ± std)
Persistence	132.09/177.84/1035.96; 448.63 ± 415.72	41.11%/44.27%/34.00%; 39.79% ± 4.29	2.81%/3.07%/0.98%; 2.29% ± 0.93%
GCRN	108.55/103.20/1422.58; 544.77 ± 620.71	36.70%/36.08%/38.76%; 37.18% ± 1.14	64.98%/67.25%/65.27%; 65.83% ± 1.01%
LTGCN	122.33/123.87/1763.79; 670.00 ± 773.43	35.04%/36.49%/46.52%; 39.35% ± 5.11	61.96%/63.22%/64.18%; 63.12% ± 0.91%
GTGCN	122.57/152.52/1838.79; 704.63 ± 802.07	38.41%/48.59%/51.72%; 46.24% ± 5.68	58.15%/59.80%/63.55%; 60.50% ± 2.26%

### Ablation variants results

The ablation study confirms that all variants contribute meaningfully to performance. Table 10 presents ablation results for Spain dataset. All comparable variants are matched within 1% budget with count of parameters that ranges between 267K-270K. Table 11 reports ablation results for Top-40 subset in Brazil dataset. All comparable variants are matched within 11% budget with count of parameters that ranges between 66K-73K.

**Table 10:** Ablation study results for Spain dataset comparing LTGCN variants

Variant	#Params	RMSE	SMAPE (%)	MDA (%)
Spatial-only (no transformer)	267,565	1083.98	39.21%	54.31%
Temporal-only (no GCN)	269,869	1776.30	46.58%	65.35%
LinearTGCN (linear + GCN)	267,489	682.92	24.74%	72.52%
No-fusion (concat, no attn)	268,209	1819.12	51.63%	60.56%
Full LTGCN	269,045	1763.79	46.52%	64.18%

**Table 11:** Ablation study results for the top-40 subset in Brazil dataset comparing LTGCN variants

Variant	#Params	RMSE	SMAPE (%)	MDA (%)
Spatial-only (no transformer)	68,141	3882.20	105.76%	40.67%
No-fusion (concat, no attn)	72,931	3873.66	87.47%	47.16%
LinearTGCN (linear + GCN)	68,179	3933.80	84.32%	49.68%
Full LTGCN	69,031	3873.63	83.47%	52.02%
Temporal-only (no GCN)	65,657	3872.37	**81.90%**	50.12%

**Table 12:** Parameters, batch size, epoch time, and attention complexity for each model on Spain dataset

Model	#Params	Batch size	Epoch time (s)	Attention complexity
Persistence	0	–	0.00	*O*(1)
GCRN	250,849	32	0.46	$O(N \cdot T)$
LinearTGCN	267,489	32	0.03	$O(N \cdot T)$
Graph WaveNet	247,483	32	1.02	$O(N \cdot T)$
LTGCN	269,045	32	0.32	$O(T^2)$ per node
GTGCN	224,609	16	0.18	$O((TN)^2)$

### Summary of key results

Several trends emerged from our experiments, which we explore in greater detail in the Discussion section. We observed a substantial performance gain across all models after cleaning the data, highlighting the importance of data quality. The LinearTGCN result (best on all metrics in Spain) reports that the GCN spatial encoder is the primary driver of performance gains over GCRN; not the attention mechanism. Self-attention appears marginally useful for directional prediction in Brazil but provides less benefit in Spain’s cleaner dataset. In line with Zeng et al. [53], our results demonstrate that simple linear models can match or exceed Transformers on structured time series, while Transformers show a great performance on noise or unstructured datasets like Brazil dataset. Also, we found that RMSE can be biased toward high-population cities, making metrics like SMAPE and MDA more informative for regional comparisons, particularly during outbreak peaks.

A naïve persistence forecast carrying the last observation forward achieves near-zero directional accuracy (MDA $= 0.98$ and fold-average $2.29\%$ ± $2.3\%$ in Spain), confirming that epidemic trend detection requires genuine temporal modeling. The results show that while SMAPE between models differs by only a few percentage points, the models are significantly better at directional prediction than the naïve baseline. On high RMSE variance, Fold 3 always tests against a COVID-19 peak window, producing RMSE 3–4 times higher than folds 1–2; indicating that fold 3 covers a later, higher-wave period.

Compared to Brazil, Spain’s uniformly reported dataset required no data cleaning, enabling models to perform optimally out of the box. GTGCN performed comparably on directional accuracy (MDA) but was less effective on RMSE and SMAPE, raising questions about its added complexity in homogeneously reported datasets. A deeper discussion of these results follows in the next section.

## Discussion

This section synthesizes the results from both Brazil and Spain to draw broader conclusions about the predictive performance of spatio-temporal models in epidemic forecasting. We analyze the influence of data quality, architectural decisions (recurrent vs. Transformer-based), and spatial modeling approaches (GCN vs. global attention) on key evaluation metrics. Insights from the ablation study are integrated to understand model behavior under different configurations and input regimes.

Notably, the LinearTGCN variant which replaces the self-attention temporal encoder with a simple linear lag projection; achieves the best SMAPE (24.74%) and MDA (72.52%) on the Spanish dataset, outperforming the full LTGCN with attention. While, in the Top-40 subset of Brazil dataset, LTGCN slightly outperforms LinearTGCN and other variants with RMSE(3873.63) and SMAPE(83.47%). This suggests that in consistently-reported epidemiological datasets, the primary benefit derives from the GCN spatial encoder and the fusion mechanism, not from attention. Additionally, the full LTGCN achieves stronger directional gains on the noisier Brazilian data, where attention may help smooth irregular reporting artifacts. Our results reveal that while simple linear architectures achieve competitive or superior performance on structured time series, Transformers exhibit a distinct advantage on unstructured or high-noise datasets.

Although global attention mechanisms can theoretically capture long-range dependencies across all nodes in a graph, GTGCN fails to consistently outperform the comparable baselines on key error metrics. GTGCN achieves competitive directional accuracy (MDA) in compact, high-mobility graphs (Spain, Top-40 Brazil) but does not outperform LTGCN on RMSE or SMAPE.

In the Brazilian dataset, moving from the full set of 5300+ cities to a cleaned subset of 1305 then 40 high-quality cities led to significant gains in forecasting accuracy. This highlights the importance of preprocessing: noisy reporting, prolonged zero-case streaks, and low-variance time series can severely obscure learning signals. Unlike Brazil, the Spanish dataset exhibited uniformly reported high-quality data across all 52 provinces. No cities were filtered out and all models were evaluated on the full dataset. This consistency allowed for clearer comparisons between architectures without the confounding effects of data irregularities. A key observation from Brazil is the RMSE’s sensitivity to population size. Cities like Bras´ılia, with very high case counts, dominated the RMSE metric. In contrast, SMAPE and MDA, being scale-independent and trend-aware, offered more balanced comparisons. The results show that while SMAPE between models differs by only a few percentage points, the models are significantly better at directional prediction than the persistence baseline.

We conducted a series of ablation experiments to evaluate the impact of model depth, sequence design, and spatial encoding in epidemic forecasting. Our findings underscore the critical role of architectural decisions in both baseline GCRN and Transformer-based models. With removal of the GCN encoder and capturing only the temporal trends, peak magnitudes were often misaligned. On average, RMSE and SMAPE worsened slightly, while MDA unexpectedly increased, suggesting that GCN smoothing may trade off short-term directional precision for improved magnitude accuracy.

### Limitations and future work

#### Limitations

Despite the promising performance of our Transformer-GCN models, several limitations must be acknowledged regarding data quality, modeling design, and evaluation.

##### Data quality and reporting noise

The Brazilian dataset contains irregular reporting patterns, such as prolonged zero-case streaks followed by large spikes and occasional negative values. These artifacts, likely due to administrative delays or retroactive corrections, introduce noise that may affect model learning. Although filtering and clipping were applied, residual distortions remain. To counterbalance this, we included a more stable dataset from Spain to evaluate robustness under cleaner reporting conditions.

##### Forecasting setup constraints

All models were trained with fixed prediction horizon of one day and city-wise z-score normalization. While these choices simplify the learning task, they may limit the capture of long-range temporal dependencies and introduce distortions during highly non-stationary phases.

##### Evaluation limitations

Although RMSE was the primary evaluation metric, it is sensitive to population size and does not fully capture the alignment in outbreak timing. We supplemented this with SMAPE and MDA, yet qualitative aspects, such as trend sharpness and peak timing, remain partially unquantified. Our models produce deterministic predictions without calibrated uncertainty. Epidemic decision-making systems increasingly require probabilistic forecasts (WIS, prediction intervals) [54]. Extending LTGCN/GTGCN to produce distributional outputs is an important direction for future work.

#### Future work

Addressing the above limitations suggests promising directions for future research:

- **Hybrid architectures:** Combining Transformers with sequential smoothing units such as GRUs [40] may better balance long-range context and local continuity.
- **Sparse attention for scalability:** Employing sparse or linear attention mechanisms [2, 55] can reduce computational cost and enable longer forecasting horizons.
- **Multi-modal temporal fusion:** Integrating auxiliary signals such as climate data, sentiment trends, or containment measures could improve robustness and interpretability.
- **Probabilistic Evaluation:** All models produce point forecasts; probabilistic evaluation (WIS, interval scores) is left for future work.
- **Transfer learning:** Leveraging cross-region training and fine-tuning may enhance generalization to data-scarce or unseen locations.

Pursuing these avenues will strengthen the interpretability, accuracy, and scalability of spatio-temporal DL systems for epidemic forecasting and related real-world applications.

## Conclusion

This work investigated the effectiveness of advanced spatio-temporal DL architectures for regional epidemic forecasting, focusing on hybrid models that combine GNNs with Transformer-based temporal encoders. Evaluated across COVID-19 datasets from Brazil and Spain, these models were assessed for their ability to capture both spatial dependencies and complex temporal dynamics.

Our findings highlight that LTGCN and GTGCN show a great performance comparing to other baselines especially in noisier datasets. Remarkably, attention-models is not always the answer, the experiments reports that LinearTGCN variant outperforms all models in Spain dataset, which explains that the GCN spatial encoder is a primary factor of performance gains over GCRN, especially in structured datasets. Self-attention appears marginally useful for directional prediction in noisy and unstructured dataset like Brazil. GTGCN achieved competitive results in terms of RMSE and SMAPE but incurred substantial computational overhead due to its quadratic attention complexity. Additionally, we demonstrate the importance of rigorous preprocessing strategies, including population normalization, data quality filtering, and spatial backbone construction. Integrating GCN-based encoders improved forecast calibration across regions, particularly in capturing the timing and magnitude of outbreak peaks.

Overall, our approach contributes to a flexible and scalable forecasting framework suitable for diverse public health applications. By fusing temporal attention, spatial graph reasoning, and robust data handling, our models form a strong foundation for next-generation epidemic forecasting systems.

## Data Availability

The source code is available at (https://github.com/mobyous/LTGCN-GTGCN). The processed datasets generated in this work are available at (https://doi.org/10.5281/zenodo.17992015) under restricted access. Access is granted for research purposes upon reasonable request.
